# Moments of meeting: A case study of Shared Reading of poetry in a care home

**DOI:** 10.3389/fpsyg.2022.965122

**Published:** 2022-09-27

**Authors:** Thor Magnus Tangerås

**Affiliations:** Kristiania University College, Oslo, Norway

**Keywords:** Shared Reading, dementia, moments of meeting, intersubjectivity, poetry

## Abstract

There is a growing research interest in the value of participative arts-based strategies for enhancing wellbeing amongst adults living with dementia. One such intervention, centred around literature, is the group activity called Shared Reading. The purpose of this case study of weekly Shared Reading sessions of poetry in a care home in Merseyside is to investigate instances of how participants with mild to moderate dementia collaborate in processes of meaning-making that allow them shared experiences of *being moved by poetry*. An under-thematised aspect of psychological wellbeing is the capacity for being moved and for sharing such moments. This article addresses the following question: how can the specific multimodality of the text (participants have a copy of the text before them, the poem is read aloud and there may be use of non-verbal aids) in the Shared Reading model help to bring about such experiences? Using Stern’s concepts of Now Moments and Moments of Meeting, this case study discusses various instances of unpredictable, surprising and spontaneous intersubjective moments between participant and poem, participant and reader leader, participant and staff, participant and relative.

## Introduction

In recent years “there has been a growing awareness that art and aesthetics have an important role to play in delivering healthcare as well as a reappraisal of the associations between the arts and society in general” (Zeilig et al., 2018, p 135). Concomitantly, there has been increased research interest in the value of arts-based strategies for enhancing wellbeing amongst adults living with some form of dementia. Many subtypes of dementia have been defined, each with a different pathway or process (Stephan and Brayne, 2010), but in general terms dementia is characterised by progressive decline in cognition of sufficient severity to interfere with activities of daily living (Knopman et al., 2001). In their review Beard (2011) differentiate between two distinct orientations underpinning participative arts interventions: art as therapy, and art as activity. The former has a biomedical focus, emphasising clinical outcomes, such as the reduction of the behavioural and psychological symptoms of dementia, whereas the latter prioritises person-centred outcomes, such as providing a source of pleasure and enhancing quality of life. The emerging consensus is that interventions using music, dance, drama, visual arts, and poetry provide positive outcomes for people with dementia (Selberg, 2015). Concretely, they have been found to improve subjective wellbeing (Johnson et al., 2015), learning (Bannan and Montgomery-Smith, 2008), the opportunity to exercise autonomous choice (Melhuish et al., 2017), and enhanced quality of life (Hannemann, 2006).

These findings, however, remain tentative. In a critical review of participative arts interventions, Zeilig et al. (2014) emphasise that adequately evaluating complex interventions such as participative arts projects is difficult as they involve interrelated variables, confounding factors, and a range of possible outcomes. Specifically, as regards poetry interventions, they conclude that “despite the anecdotal evidence that many projects use poetry and creative writing with people living with dementia, studies evaluating the efficacy of these interventions (especially from the point of view of the PWD) are scant and work examining the applications of poetry using rigorous, robust methodologies is rather sparse.” (p. 23). When it comes to Shared Reading, however, such rigorous investigations into the impact and effect of reading literature with people with dementia have been undertaken. Billington et al. (2013) have studied Shared Reading conducted in care homes, hospital wards and day centre. Using the Neuropsychiatric Inventory Questionnaire (NPI-Q) to assess staff views of changes in dementia symptom severity for participants in the Shared Reading groups, they found a significant reduction. This effect was predicated not only on enhancement of listening, memory and attention, but also on enjoyment, meaningfulness and renewed sense of personal identity. Furthermore, Longden et al. (2016) carried out a RCT on Shared Reading for adults living with dementia. Quality of Life was measured and the study found that compared to the control condition, the positive effects of Shared Reading on QoL were maintained once the activity ended.

According to Dowrick et al., Shared Reading “not only harnesses the power of reading as a *cognitive* process, but also acts as a [...] socially coalescing presence, allowing readers a sense of subjective and shared experience.” (Dowrick et al., 2012, p. 16). It is this power of Shared Reading to create meaningful interaction and a coalescing presence I wish to explore in this article. The purpose of this case study of the use of poetry in weekly Shared Reading sessions in a care home in Merseyside is to investigate instances of how participants with mild to moderate dementia collaborate in processes of meaning-making that allow them shared experiences of *being moved by poetry*. An under-thematised aspect of psychological wellbeing is the capacity for being moved and for sharing such moments. This article addresses the following question: how can the specific multimodality of the text (each reader has the text before them, the poem is read aloud and there may be non-verbal props in the form of, e.g., photographs related to motives in the poem) in the Shared Reading model create moments of meeting?

## Method and theory

The study is inspired by ethnomethodology’s focus on the processes of meaning-making that are present in social interactions (Garfinkel, 1967). Thus, the process of meaning-making of the literary text is embedded in a group setting, and the group is formed and created through this practice. There is also an action research motivation underlying the study, which “aims at changing three things: practitioners’ practices, their understandings of their practices, and the conditions in which they practise” (Kemmis, 2009, p. 463), and which is thus both practice- based and practice-led (Candy, 2006). This case study is based on weekly sessions of Shared Reading in a care home in Merseyside carried out over a period of 12 weeks. The average number of participants was 7–8, ranging from 5 to 12. Some of the participants showed only mild symptoms of dementia, while others had quite severe cognitive impairments. None of the sessions were audio-recorded, so that the empirical material consists of post-session notes made by me as both reader-leader and researcher. The real names of participants have been altered, so that pseudonyms are used throughout, and identifiable information has been excluded. Sessions were conducted in a secluded corner of an open lounge, where we would sit in comfortable chairs forming a neat circle.

All case studies spring from the need to develop in-depth understanding through empirical research of a contemporary phenomenon, based on a small set of instances and complex conditions set in their real-world contexts (e.g., Bromley, 1986). This case study is both descriptive and explanatory (Zeilig et al., 2018) in that it seeks to describe what happens/appears in sessions of Shared Reading, in light of a theoretical framework borrowed from intersubjective psychotherapy. Previous studies of Shared Reading have identified “breakthrough moments” (Davis et al., 2016; Gray et al., 2016; Longden et al., 2016)–moments in which cognitive and affective turning-points take place that may have lasting benefit for the participant, when

The literature seems to get through to participants, and participants seem to experience a change or awakening taking place beneath the level of intention and beyond the general norms of response or opinion. Evidence that something significant had happened, through a change in the perception of reality, was sought through analysis of the transcripts. These moments, themselves specific and emotionally powerful, were often shown to the participants at interview, to test their validity and significance (Davis et al., 2016).

Such breakthrough moments are understood as intrapsychic events that may be communicated to the group, but can also be private events not shared in the group with the reader leader or other members. For an in-depth discussion of such a private breakthrough moment that had long-lasting life-changing effects, (see Tangerås, 2020). As such, they are similar to what Stern et al. term “now moments”, characterized by elements of surprise, spontaneity, unpredictability and heightened affect:

These moments are pregnant with an unknown future that can feel like an impasse or an opportunity. The present moment becomes very dense subjectively as in a “moment of truth”… a “now” moment is an announcement of a potential emergent property of a complex dynamic system. Although the history of its emergence may be untraceable, it is prepared for with fleeting or pale prior apparitions, something like a motif in music that quietly and progressively prepares for its transformation into the major theme. Still the exact instant and form of its appearance remain unpredictable (Stern et al., 1998, p. 912).

Such “now moments” may turn into intersubjective moments of meeting. If it is mutually recognized and grasped, the opportunity becomes “lit up” as an affectively rich “moment of meeting” in which “each partner has actively contributed something unique or authentic of his or herself as an individual” (p. 913):

The key concept, the “moment of meeting”, is the emergent property of the “moving along” process that alters the intersubjective environment, and thus the *implicit relational knowing.* In brief, *moving along* is comprised of a string of “present moments”, which are the subjective units marking the slight shifts in direction while proceeding forward. At times, a present moment becomes “hot” affectively, and full of portent for the therapeutic process. These moments are called “now moments”. When a now moment is seized, i.e., responded to with an authentic, specific personal response from each partner, it becomes a “moment of meeting”. This is the emergent property that alters the subjective context (Stern et al., 1998, pp. 909–910).

This kind of emergent property can only arise if the moving along occurs within a context that is rule governed by an established technique that is (implicitly) well understood by the interactants. Affective sounds, head nods, eye contact, and other nonverbal communication are often observed in such moments, followed by brief pauses of “open space” where each sits with and takes in what just occurred. Stern gives two concrete examples: looking at someone in the eyes who is looking at you, and taking a deep breath while talking to someone (Stern, 2004, p. xv). According to Stern, moments of meeting constitute “the key moments of change in psychotherapy” (Stern, 2004, p. xi).

The Boston Process of Change Study Group developed the theory based on detailed observations of “implicit relational knowing” in mother-infant interactions (Stern et al., 1998) and the theory has been applied principally to psychotherapy, but increasingly also to other contexts of pedagogy and learning. For instance, Schneider and Keenan find that “the intersubjective space of the classroom can provide students with experiences of being known and “moments of meeting” which can result in transformative learning” (Schneider and Keenan, 2015, p. 1). There are four distinct phases: moving along, now moments, moments of meeting and open spaces. These are directly applicable to Shared Reading. Thus we may say that “moving along” corresponds to the activities of “getting into” and “staying with” the poem in Shared Reading–the normal procedures of asking questions in relation to the poem, commenting on it or responding to other members’ comments. This “moving along” may be punctuated by “now” moments, akin to “breakthrough moments”. If these moments are fully met and responded to by either the reader leader or another participant, there may be a moment of meeting, which subsequently segues into an “open space” of stillness and change of affect. In the following I will, through eight vignettes, explore such moments of meeting.

## Moments of meeting

### An unexpected procedural rupture marked by co-creativity

The first moment of meeting occurred at the very end of the initial session. We had been reading and talking about Philip Larkin’s poem Coming. I had chosen it on the basis of personal liking as well as having witnessed it being used with success by another reader leader in a different care home. The poem’s theme is resonant for the participants in that it treats of specific childhood memories, and also of implicit affective knowing that may not be verbalizable: “…. And I, whose childhood / Is a forgotten boredom, / Feel like a child /Who comes on a scene / Of adult reconciling, / And can understand nothing / But the unusual laughter, / And starts to be happy”. However, although the discussion was productive and touched upon memories of both spring and childhood, the session may be understood to have been marked by a “moving along”, in that no apparent “now moments” occurred. It was pleasant and several members contributed actively – questioning the metaphor of foreheads of houses being bathed. But then at the end something unprecedented happened. It is customary to round off the session by a final reading aloud of the poem, ideally a reading which assimilates and is resplendent with all the various meanings we have touched upon during the dialoge. I proceeded to end the talk, and said “so, let us read the whole poem one final time.” As I started to read the first words, “On longer evenings…,” the participants joined in, one after the other, building to a chorus as we all solemnly but with enjoyment read it aloud together. An entirely surprising, unpredictable and affect-laden experience, and the participants could see that they had greatly surprised me, and several smiles were exchanged. From this moment of meeting, an “open space” was clearly opened up, as we just sat in silent tranquil enjoyment for a long while afterwards. How this arose I will never know. Perhaps it stemmed from a practice common during their school days, which they simply assumed was what we were meant to do. Alternatively, they took my words at face value: “let *us* read it.” What is more important is that this moment created a change of procedure and established a group culture, as on each subsequent occasion we would repeat the chorus at the session’s close. It was an important instance of co-creation (Zeilig et al., 2018), of everybody contributing toward a socially coalescing, implicit relational knowing.

### Acknowledged discovery of performative skills in participant

The following week I opted for Christina Rossetti’ sonnet *Remember.* This may have been a bold choice, as it encourages participants to envisage themselves saying goodbye to a loved one or indeed to life itself. But I knew other reader leaders had used it in care homes. Once again the now moment was connected to the reading aloud aspect. This time, however, it was the surprise discovery of the declamatory abilities of one of the participants that really stood out. The standard procedure in Shared Reading is for the reader leader to read the poem aloud once, and then leave a pause, and if no one comments then offer to read it again, inviting the participants to have a go. At first it did not seem as if anyone would accept the invitation, but then I happened to glance at Katherine, who until this point had been very quiet and avoided eye contact with other participants. She looked up and met my gaze, but did not say anything. By now she seemed fully alert and present. I took a chance and addressed her directly, inviting her to read aloud. Such direct requests can be risky, and in general I tend to avoid them, but I got the sense that she wanted me to ask her. She hesitated for a few seconds, then launched into the most beautiful rendition of the poem, mellifluous, sonorous and rhythmic. Evidently, her reading prompted immediate recognition from the group, as members exclaimed, “ah, that was wonderful” and “thank you.” Katherine seemed both energized and proud afterwards – a clear now moment. I consider it also as a moment of meeting in that her skill was acknowledged by us. We would remember this, so that next time, when reading Wordsworth’s Composed Upon Westminster Bridge, participants actively encouraged her to open the session by reading it, and I conceded that she would perform it better than I could. As such, it marked a rupture from normal procedure where the reader leader starts by reading the poem aloud. It was a moment that altered both of us. It transpired that she had been a teacher by profession, and also engaged in amateur theatre groups in her younger years.

### I must go down to the seas again

Many people in Merseyside have a connection to the sea, so I thought it would be good to include John Masefield’s poem *Sea Fever.* The poem gives voice to a universal longing and should therefore appeal to the whole group. Our subsequent discussion proved me right in this, as participants reminisced about family members or days gone by. One of the participants, Henry, told us of his time working in the port, and reflected upon how much society has changed since then. But again, the now moment was related to the act of reading the poem aloud. In another article I have thematized the *double modality* of Shared Reading, the affordances made possible by participants having a copy of the text to read and at the same time being able to listen to the text being read aloud (Skjerdingstad and Tangerås, 2019). In Shared Reading in care homes, this may be expanded to a multimodality (Jewitt, 2007; Kress, 2010). It can be very productive to bring along photos of images related to the poem, for instances pictures of birds (such as a thrush in the case of Larkin’s poem) so that participants more easily and vibrantly can envisage the world of the poem. This time, in leading the discussion, as I repeated several of the stanzas of this poem–which is marked by a series of repeated conjunctives (“and,” “and”) and has a very strong rocking rhythm reminiscent of waves–I would use nonverbal gestures to evoke the imagery. Using exaggerated rhythmic hand gestures and gently swaying my upper body from left to right, I attempted to embody the motion of the waves and the sounds of the sea. This was apparently successful: one participant, Margareth, seemingly without being aware of it, starting to move along in imitation of my gestures. Afterwards, I remarked to her: “you enjoyed that poem, did not you, Margareth?” and she nodded vigorously. The reason I interpret it as a now moment turning into a moment of meeting, is that she was subsequently more active in our discussion of the poem than she had been in previous weeks. She said she could vividly hear “the cry of the gulls, and the splashing of the waves and the feel of the salty wind on my face,” thus reiterating not just the images but the repetitions of the conjunctive “and.”

### The reluctant relative and the fear of being upset

Just as we were about to begin a session, we were interrupted as the husband of one of the participants, Jill, came to visit her. I explained to him about the Shared Reading and that it was open to all, but he was very skeptical and expressed concern lest his wife get “upset” by the poem. Instead of contradicting him, I simply invited him to take part so that he could look after her in case she did get upset. However, as he could see that Jill was contentedly listening to the reading, and also chipping in with some comments during the discussion of the poem (Shakespeare’s Sonnet XXX), he started to relax. Jill seemed very pleased and proud to show him what she was part of, and in the way he looked at her afterwards, I could sense that he acknowledged this, and that there was a moment of meeting between the two of them. They took a copy of the poem away with them, as he wheeled her back to her room afterwards. It made me reflect on the word “upset.” Although it has a negative valour and is connected to unwanted feelings, it is closely connected to “now moments”, in that it represents a rupture which opens up the possibility of repair, if met with empathy.

### To those first feelings that were born with me

A now moment may come as a rupture of an interaction pattern or a set way of doing things. One of the regular group members, Elsie, would, as soon as we had finished reading a poem aloud, invariably exclaim, regardless of the poems content or tone: “aw, that’s nice.” This habitual response was probably a collaborative effort, to show her appreciation. But she seemingly could never follow up on that remark by entering into exploration of the words on the page. The first time she said it, I simply nodded in acknowledgment and smiled, hoping for more. The second time I asked whether there was something particular that she liked about the poem. Perhaps that was too challenging, because she became flustered and bewildered, as if she had been rebuked. On other occasions, someone else would start talking afterwards. On this particular occasion, however, something happened. The poem in question is a rather difficult poem by Emily Brontë, “Often rebuked, yet always back returning”. (Curiously, the opening two lines seemed to address this particular sensation: “Often rebuked, yet always back returning / To those first feelings that were born with me.”) After Katherine had read the poem for us, there was a pause, and Elsie made to say something. I expected the usual comment, but this time she instead proceeded to read aloud the penultimate stanza of the poem:

I’ll walk where my own nature would be leading:

It vexes me to choose another guide:

Where the gray flocks in ferny glens are feeding;

Where the wild wind blows on the mountain side.

It was very evident that this had made an impact on her, as she was straining to articulate something. “My parents and I, I remember… Us going hillwalking. Going to the mountains. It was windy, the nature, lovely.” She wanted to say more, but stopped. It was clear that it was the concrete stanza that had elicited a specific episodic memory. Although not very detailed, the memory evidently had a specific feel to it, as her tears welled up and she looked up into the distance as if she could see the mountain top. So I repeated the last line of the stanza whilst getting eye contact, and she nodded as if I had understood. The importance of increased specificity of memories has been demonstrated by Serrano et al. (2004); using Autobiographical Retrieval Practice, they found that older adults showed decrease in hopelessness and increased life satisfaction when enabled to retrieve specific memories from childhood.

### Dear John

The Friday following Remembrance Day we read a poem by Robert Herrick, “To Anthea who may command him anything.” The group all agreed it was a lovely poem. One participant suggested it might be a letter from a soldier to his girlfriend back home. Another member picked up on this idea, raising the question whether it could have been a Dear John letter. Then Henry divulged a very significant biographical memory: he had been a soldier, and had been very much in love, “but when I got back home she was seeing someone else.” And then he told us he had never got over it and had never married. The participant sat next to him reached out to him and said “that is really sad,” while gently touching his shoulder. And then someone else spontaneously read out loud the third stanza:

Bid that heart stay, and it will stay,

To honour thy decree;

Or bid it languish quite away,

And ‘t shall do so for thee.

His recollection sprung out of the poem, and his disclosure elicited genuine empathy from several other participants. Furthermore, staff told him afterwards that they did not know this about his life, and felt that they had got to know him better. I consider this an instance of a moment of meeting between poem and participant, between participants, and between participant and staff. The suggestion that the poem was about a soldier writing home was not an obvious one to me. It made me speculate about the following: During our previous session, on Remembrance Day, we had read MCMXIV by Larkin, which famously ends:

Never such innocence,

Never before or since,

As changed itself to past.

Without a word – the men

Leaving the gardens tidy,

The thousands of marriages,

Lasting a little while longer:

Never such innocence again.

Could it be that the memory of this poem was carried over into the reading of the Herrick poem, that the context for Larkin’s poem resonated with Herrick’s love poem? As if there had been a moment of meeting between the two poems themselves.

### I hear it in the deep heart’s core

Upon reading Yeats’ famous poem The Lake Isle of Innisfree, several members said they liked and enjoyed it. One of the new members spoke a lot, sometimes engaging with the poem, other times veering off into off-topic reminiscences and confused thoughts about present and family relations. Henry was the first to question the poem, wondering “how can you possibly hear the water lapping when you are stood in a road in the midst of city noise?” Another participant suggested that he could still hear it inside him, in his heart. Then Elsie read a line out loud: “And I shall have some peace there, for peace comes dripping slow.” Pat then commented that it said *some* peace, that maybe there was still unrest, disquiet in this person. Henry, having pondered the meaning of the last stanza, then seemed able to relate to their comments, offering the suggestion that the person wanted to get away from his past, and that sometimes the dream is a means of escape. After musing for a few seconds, he thought perhaps it could be somebody who has lost what he has. I think that is quite a profound reading. In this poem’s repetition of “I will arise and go” there is a clear allusion to the (King James) Bible, to the parable of the Prodigal Son who indeed loses all he has and goes back home. I am not saying that Henry was cognizant of this allusion, but it was his interpretation that made me connect the poem to the parable, hence he expanded my understanding of the poem. When I acknowledged his interpretation, saying I had never thought of that, he gave me a little smile.

### After the end

The final moment I will relate is an astonishing one. As I walked through the tv-lounge on my way to the corner where the group assembles, I noticed an old lady sat slumped in front of the telly. She did not look as if she would be able to join in the Shared Reading, as she was unable to raise her head, and when I went up to talk to her, a member of staff said that she was too ill to take part. However, when I asked her whether she might like to listen to a poetry reading, she responded affirmatively. Maeve was duly placed within the circle of participants, but was unable to hold the sheet of paper containing the poem Rainbow Children by Seamus Heaney. Kindly, Pat, the participant next to her, assisted her by holding it up for her to see. Frankly, I expected little from Maeve by way of active participation. But in this I was wrong. We read and discussed the poem. We spent some time trying to figure out the details of the setting, how the children could be eye-level with the white cups. We read it through several times.

I felt that the session went ok, although perhaps they found it harder to engage with this poem than I expected. They did, however, engage in some complex thinking about the relationship between then and now, the individual and time, elicited in particular by the line “we were small and thought we knew nothing.” Only after we had finished reading it aloud together one final time did Maeve speak. In a barely audible voice she related how God had talked to her while she was walking to school one morning when she was “only little.” And that she had told no one about this, not daring to tell even her mum. This was brought about by the religious imagery of the poem’s ending, where the raindrops are “full with the light of the sky” and the children “could stream through the eye of a needle.” In terms of “open spaces”, I think the sharing of this remarkable biographical incident opened up the poem for us all.

## Conclusion

“O body swayed to music, O brightening glance,

How can we know the dancer from the dance?”

These vignettes describe eight moments that I understand to be now moments that became moments of meeting. Extrapolating from a psychotherapy context, we may say that insofar as the now moment is sustained and shared between two or more subjectivities–reader leader and participant, between participants or relatives/members of staff–as long as the other is able to grasp the moment and explore it, the possibility of a moment of meeting emerges. In a qualitative study of a variety of therapists from different orientations, Duarte et al., 2022 found that.

Moments of meeting were described by all therapists as co-constructed moments that take place during the process through therapist-patient interaction. These moments are spontaneous and lived as a genuine and unplanned experience. They are creative, unique and singular experiences that produce feelings of emotional synchrony that can take different forms and levels of intensity. (p. 516).

Furthermore, all therapists interviewed agreed that these moments have a very powerful effects for the therapy process and that they “also have a long-term effect that expresses later during the process. (p. 517),” thus contributing to long term changes. Did the moments of meeting I have described lead to lasting change? I have no way of knowing, but they may have effected change in the short term. One thing is for certain, though: They have had a lasting impact on me and constitute some of the strongest experiences I have had as a reader leader. Duarte et al. suggest that.

Another interesting area for further research is the effect that MoM may have on the therapists and if it is possible to understand them as therapeutic for the therapist as well as the patient. We hypothesize that through these reminiscences and experiences we can also see a profound psychological effect on the therapist that may influence and reinforce their work and their role. (p. 522).

I think this is a topic that would be interesting to explore in the context of participative arts and bibliotherapy: how is the practitioner changed by her practice?

## Data availability statement

The datasets in this article are not readily available because the empirical material consists of field notes. The names of all participants have been altered. Pseudonyms are used throughout. No specific biographical information about participants’ backgrounds is included. Requests to access the datasets should be directed to thormagnus.tangeras@kristiania.no.

## Ethics statement

Ethical review and approval was not required for the study on human participants in accordance with the local legislation and institutional requirements. Written informed consent for participation was not required for this study in accordance with the national legislation and the institutional requirements.

## Author contributions

The author confirms being the sole contributor of this work and has approved it for publication.

## Funding

This study was supported by Kristiania University College.

## Conflict of interest

The author declares that the research was conducted in the absence of any commercial or financial relationships that could be construed as a potential conflict of interest.

## Publisher’s note

All claims expressed in this article are solely those of the authors and do not necessarily represent those of their affiliated organizations, or those of the publisher, the editors and the reviewers. Any product that may be evaluated in this article, or claim that may be made by its manufacturer, is not guaranteed or endorsed by the publisher.
